# Deep transfer learning for detection of breast arterial calcifications on mammograms: a comparative study

**DOI:** 10.1186/s41747-024-00478-6

**Published:** 2024-07-15

**Authors:** Nazanin Mobini, Davide Capra, Anna Colarieti, Moreno Zanardo, Giuseppe Baselli, Francesco Sardanelli

**Affiliations:** 1 Department of Biomedical Sciences for Health, Università degli Studi di Milano, Milan, Italy; 2 Postgraduation School in Radiodiagnostics, Università degli Studi di Milano, Milan, Italy; 3 Radiology Unit, IRCCS Policlinico San Donato, Via Morandi 30, 20097 San Donato Milanese, Italy; 4 Department of Electronics, Information, and Bioengineering, Politecnico di Milano, Milan, Italy; 5 Present Address: Lega Italiana per la lotta contro i Tumori (LILT) Milano Monza Brianza, Milan, Italy

**Keywords:** Artificial intelligence, Breast arterial calcification, Cardiovascular diseases, Deep learning, Mammography

## Abstract

**Introduction:**

Breast arterial calcifications (BAC) are common incidental findings on routine mammograms, which have been suggested as a sex-specific biomarker of cardiovascular disease (CVD) risk. Previous work showed the efficacy of a pretrained convolutional network (CNN), VCG16, for automatic BAC detection. In this study, we further tested the method by a comparative analysis with other ten CNNs.

**Material and methods:**

Four-view standard mammography exams from 1,493 women were included in this retrospective study and labeled as BAC or non-BAC by experts. The comparative study was conducted using eleven pretrained convolutional networks (CNNs) with varying depths from five architectures including Xception, VGG, ResNetV2, MobileNet, and DenseNet, fine-tuned for the binary BAC classification task. Performance evaluation involved area under the receiver operating characteristics curve (AUC-ROC) analysis, *F*_1_-score (harmonic mean of precision and recall), and generalized gradient-weighted class activation mapping (Grad-CAM++) for visual explanations.

**Results:**

The dataset exhibited a BAC prevalence of 194/1,493 women (13.0%) and 581/5,972 images (9.7%). Among the retrained models, VGG, MobileNet, and DenseNet demonstrated the most promising results, achieving AUC-ROCs > 0.70 in both training and independent testing subsets. In terms of testing *F*_1_-score, VGG16 ranked first, higher than MobileNet (0.51) and VGG19 (0.46). Qualitative analysis showed that the Grad-CAM++ heatmaps generated by VGG16 consistently outperformed those produced by others, offering a finer-grained and discriminative localization of calcified regions within images.

**Conclusion:**

Deep transfer learning showed promise in automated BAC detection on mammograms, where relatively shallow networks demonstrated superior performances requiring shorter training times and reduced resources.

**Relevance statement:**

Deep transfer learning is a promising approach to enhance reporting BAC on mammograms and facilitate developing efficient tools for cardiovascular risk stratification in women, leveraging large-scale mammographic screening programs.

**Key points:**

• We tested different pretrained convolutional networks (CNNs) for BAC detection on mammograms.

• VGG and MobileNet demonstrated promising performances, outperforming their deeper, more complex counterparts.

• Visual explanations using Grad-CAM++ highlighted VGG16’s superior performance in localizing BAC.

**Graphical Abstract:**

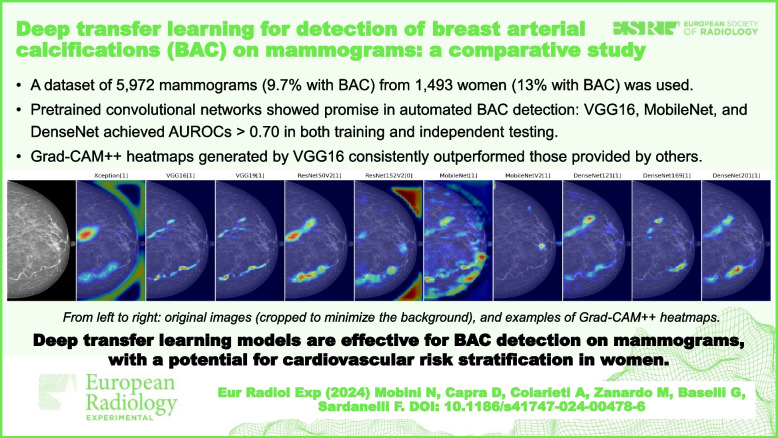

## Introduction

Cardiovascular diseases (CVD) are the primary cause of mortality and morbidity in women worldwide [1, 2]. Traditional risk scores such as the Framingham score often underestimate the risk in women, leading to missed opportunities for early diagnosis and appropriate primary prevention [3–6]. Over the past decades, breast arterial calcifications (BAC) have been advocated as a promising sex-specific biomarker of CVD to improve women’s cardiovascular stratification [7–10]. BAC are medial calcium depositions detectable as parallel line opacities on about 13% of routine mammograms [11, 12] and have been shown to be associated with an elevated hazard of CVD, independent of most conventional risk factors such as smoking [13–15]. A retrospective study by Margolies et al. [16] found a strong quantitative association between BAC and coronary artery disease. BAC scores from 4 to 12, representing a marked BAC burden, had an adjusted odds ratio of 3.2 for the presence of coronary artery calcium. Furthermore, a subset analysis in the context of a recent meta-analysis [17] conducted on studies utilizing either a 4-point scale (*n* = 4 studies) or a 12-point scale system (*n* = 3 studies) reported that mild BAC demonstrated a pooled odds ratio (OR) for coronary artery disease ranging from 1.08 (95% CI 0.42–2.75) to 2.04 (95% CI 0.82–5.05), while moderate to severe BAC showed a pooled OR ranging from 2.95 (95% CI 1.49–5.84) to 4.83 (95% CI 1.50–15.53), for studies using a 12-point scale or a 4-point scale respectively. Nonetheless, the authors of the MINERVA study (a prospective study on a cohort of over 5,000 women with a follow-up of over 5 years) [3] did not observe a quantitative association between BAC burden and hard atherosclerotic CVD events; however, they identified a threshold effect for global CVD in women over the 95th percentile of BAC. With the increasing use of mammography for breast cancer screening, BAC present an opportunity for CVD risk stratification in asymptomatic women [10, 18]. Nevertheless, their assessment is a time-consuming manual task, vulnerable to intra- and inter-observer variability [19, 20]; also, the considerable diversity of BAC’s appearance and the lack of a standard reporting guideline limited their adoption as a robust imaging biomarker in clinical practice [21, 22].

Automated methods using artificial intelligence (AI) have been recommended in the literature to overcome the intrinsic limitations of BAC detection [23–25]. The potential capability of deep learning (DL)-based approaches in extracting complex topologies of large datasets could improve the reproducibility of diagnosis while reducing radiologists’ post-processing workload. A twelve-layer deep convolutional neural network (CNN) was the first DL model developed for pixel-wise patch-based BAC detection and exhibited comparable overall performances to a human expert considering the free-response receiver operating characteristic (FROC) analysis [23]. In subsequent studies, modified versions of U-Net were explored for the similar purpose of segmenting calcified vessels and achieved higher levels of accuracy [24, 25]. However, training supervised learning models requires large-scale images with manual segmentation-level annotations, therefore still exposing the models to biases related to the inherent variability of human assessment. Nonetheless, techniques such as transfer learning from a pretrained CNN are well recognized to mitigate this issue [26, 27].

In a recent study [28] addressing automatic BAC detection and quantification, we proposed a novel transfer learning-based weakly supervised framework that effectively reduced operator dependency. By formulating the problem as a simple dichotomous classification task that only requires image-level annotations, *i.e.*, BAC or non-BAC labels instead of time-consuming pixel-by-pixel ground truth, the approach allowed estimation of calcified regions through weak supervision. Further improvements were achieved by fine-tuning a pre-trained VGG16 classification model on challenging open-source datasets, allowing the transfer of previously acquired knowledge for solving the specific BAC classification problem, without starting from scratch. Despite the study demonstrating promising results in BAC recognition, it primarily focused on optimizing VGG16 architecture, leaving the exploration of the optimal models among the state-of-the-art deep CNN networks as an open challenge subject to further research.

In this article, we compare the performance of different neural network architectures using a deep transfer learning strategy and aim to find the best models for the binary classification task of discriminating mammograms with and without BAC. The findings would assist researchers in selecting exemplary networks for detecting BAC and developing efficient tools for early CVD risk stratification, with the potential for widespread integration into clinical practices.

## Material and methods

The local Ethics Committee approved this study (Ethics Committee of IRCCS Ospedale San Raffaele; protocol code SenoRetro; approved in November 2017 and amended in May 2021) and written informed consent was waived.

### Dataset description

This retrospective single-center study included 1,493 screening mammography exams acquired using full-field digital IMS systems (Giotto IMAGE 3D or Giotto TOMO series), a dataset used in a previously published work [28]. Each examination consisted of bilateral craniocaudal and mediolateral oblique (MLO) view images of both breasts, which were reviewed by four expert readers and labeled as either BAC or non-BAC; disagreements among readers in cases of a tie were resolved by consensus. These annotated labels were encoded as the ground truth for model training, hyperparameter tuning, and performance evaluation.

Seventy percent of the exams were allocated to the training subset, 15% to the validation subset, and the remaining 15% to the testing subset. Since BAC incidence was found to be positively associated with women’s age [19], we conducted a specific data splitting strategy by defining four age classes using the BAC population’s age quartiles as thresholds, stratified splitting within each class separately to preserve BAC age distribution, and then consolidating the sub-splits into the overall corresponding subsets [28]. The training images were further randomly under-sampled reaching a BAC prevalence of 30%, to alleviate the classification bias toward the majority class of our imbalanced dataset [29, 30]. The validation and testing subsets were instead fully preserved to ensure an accurate representation of the real-world BAC prevalence.

The dataset consisted of images with various matrix sizes up to 3,584 × 2,816, depending on the compacting plates used during acquisition. Therefore, the preprocessing step involved extracting the breast regions from the dark background pixels by defining the smallest rectangular area surrounding the breast and rescaling the cropped images to a common fixed-size dimension of 1,536 × 768 pixels accepted by all the networks. Histogram analysis and Otsu’s thresholding method were used to separate the image pixels into tissue and background [31, 32]. Next, overthreshold pixel values corresponding to the breast region were normalized to reduce the intensity variation of mammographic images caused by technical or biological reasons, thus enhancing the convergence of training.

### Training setting

**Fig. 1:**
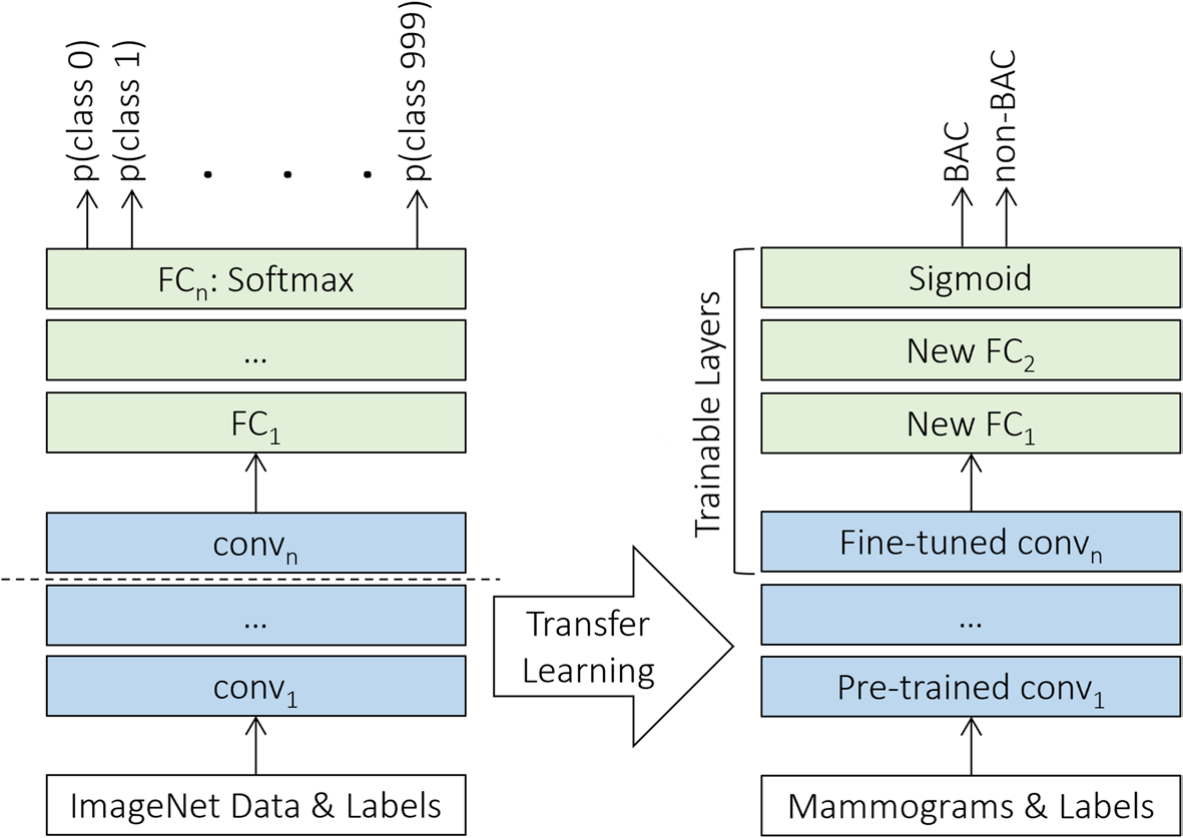
The transfer learning strategy using fine-tuning. *FC* Fully connected

Throughout the experiment, we used a total of eleven deep neural networks, namely Xception [33], VGG16, VGG19 [34], ResNet50V2, ResNet101V2, ResNet152V2 [35], MobileNet [36], MobileNetV2 [37], DenseNet121, DenseNet169, and DenseNet201 [38]. The models were previously pretrained on the ImageNet dataset, comprising more than 14 million annotated color images from 1,000 categories [39], and were publicly available through Keras Applications. Then, we implemented a uniform transfer learning strategy and a harmonized set of hyperparameters across all the networks to directly compare the performance of the various architectures, regardless of specific optimization. Since the source and our target datasets were from disparate domains, the classification layer of each was replaced with two randomly initialized fully connected layers followed by a sigmoid activation function in the output layer, as appropriate for the binary BAC classification task. For transferring knowledge, all layers in the convolutional base except the last were kept frozen with initial pretrained weights, while the rest of the deeper layers and the new classification top were fine-tuned on the mammographic dataset specifically, as illustrated in Fig. 1.

The training and evaluations were implemented using Keras and TensorFlow2 framework of Python V3.8, on a system equipped with NVIDIA GeForce RTX 3080, 10GB VRAM. Each network was retrained over 100 epochs, with a batch size of eight images limited by the available GPU memory. The Adam optimizer with an initial learning rate of 10^-3^ decayed by a cosine annealing scheduler was exploited to minimize the binary cross-entropy loss [40, 41]. Furthermore, augmentation techniques including random rotation, shifting, flipping, and zooming were applied online to the training data to avoid overfitting and improve the robustness of the classifications [42, 43]. Model checkpoint executed on the validation subset while tuning the hyperparameters and the best-performing configuration was saved at the end of each training.

### Performance evaluation

The Kolmogorov–Smirnov test was used to evaluate the normality. The continuous variables were presented by mean ± standard deviation or median and interquartile range (IQR) according to their distribution. Further, the Mann–Whitney *U* test was adopted to evaluate the age distribution disparities between the BAC and non-BAC groups, where a *p*-value less than 0.05 was considered statistically significant [44].

The overall diagnostic performance of the models against the ground truth labels was evaluated using the receiver operating characteristic curve (ROC) and area under the curve (AUC), independent of classification thresholds. Then, the true-positive (TP), true-negative (TN), false-positive (FP), and false-negative (FN) values were calculated at an optimal cutoff point, corresponding to the maximum *F*_1_ score achieved by each network on the validation dataset. The *F*_1_ score is a harmonic mean of precision and recall metrics that sought to balance the concerns of both classes in our binary classification problem:

$${F}_{1}\mathrm{ score}= \frac{2\mathrm{\ precision}\times \mathrm{recall }}{{\text{precision}}+\mathrm{recall }}=\frac{{\text{TP}}}{{\text{TP}}+\frac{1}{2}({\text{FP}}+{\text{FN}})}$$

Furthermore, we conducted a qualitative evaluation of the models’ detection and localization abilities using the generalized gradient-weighted class activation mapping (Grad-CAM++) method, which can provide a promising reader-interpretable visual explanation of the CNN models in the presence of multiple object instances within a single image, compared to the state-of-the-art [45, 46]. The technique exploited the last convolutional layer’s rich semantic and spatial information to generate a heatmap that highlighted the most informative pixels contributing to the decision-making process of the network [45, 47]. To rank these visual explanations in a somewhat quantitative manner, we assessed the Spearman correlation coefficient of the estimated calcified region delineated through thresholding of the heatmaps [28], against the corresponding manual measurements of BAC lengths previously measured in a subgroup of BAC exams with MLO views [19].

## Results

### BAC detection

The ground truth annotation indicated the presence of BAC in 194/1,493 women (13.0%) and 581/5,972 images (9.7%). The participants’ median age was 59 years (interquartile range [IQR] 52−68), where women with BAC had a significantly higher median age of 70.5 years (IQR 60–73) compared to non-BAC women (median age 57, IQR 52–65, *p* < 0.001). Following data partitioning, 410 women were assigned for training (1,640 views, including 398 BAC), 222 for validating (888 views, including 89 BAC), and 229 for testing (916 views, including 94 BAC). The training subset BAC prevalence was artificially increased by random under-sampling to address the class imbalance bias. Table 1 presents the final composition of the subsets. The patient-level data splitting prevented biases that could arise from allocating different views of an individual to different subsets.

**Table 1:** Breast arterial calcifications (BAC) and non-BAC distributions over the subsets

	Number of mammographic images		
	BAC	Non-BAC	Total
Training	398	1,242	1,640
Validation	89	799	888
Testing	94	822	916

*BAC* Breast arterial calcifications

**Fig. 2:**
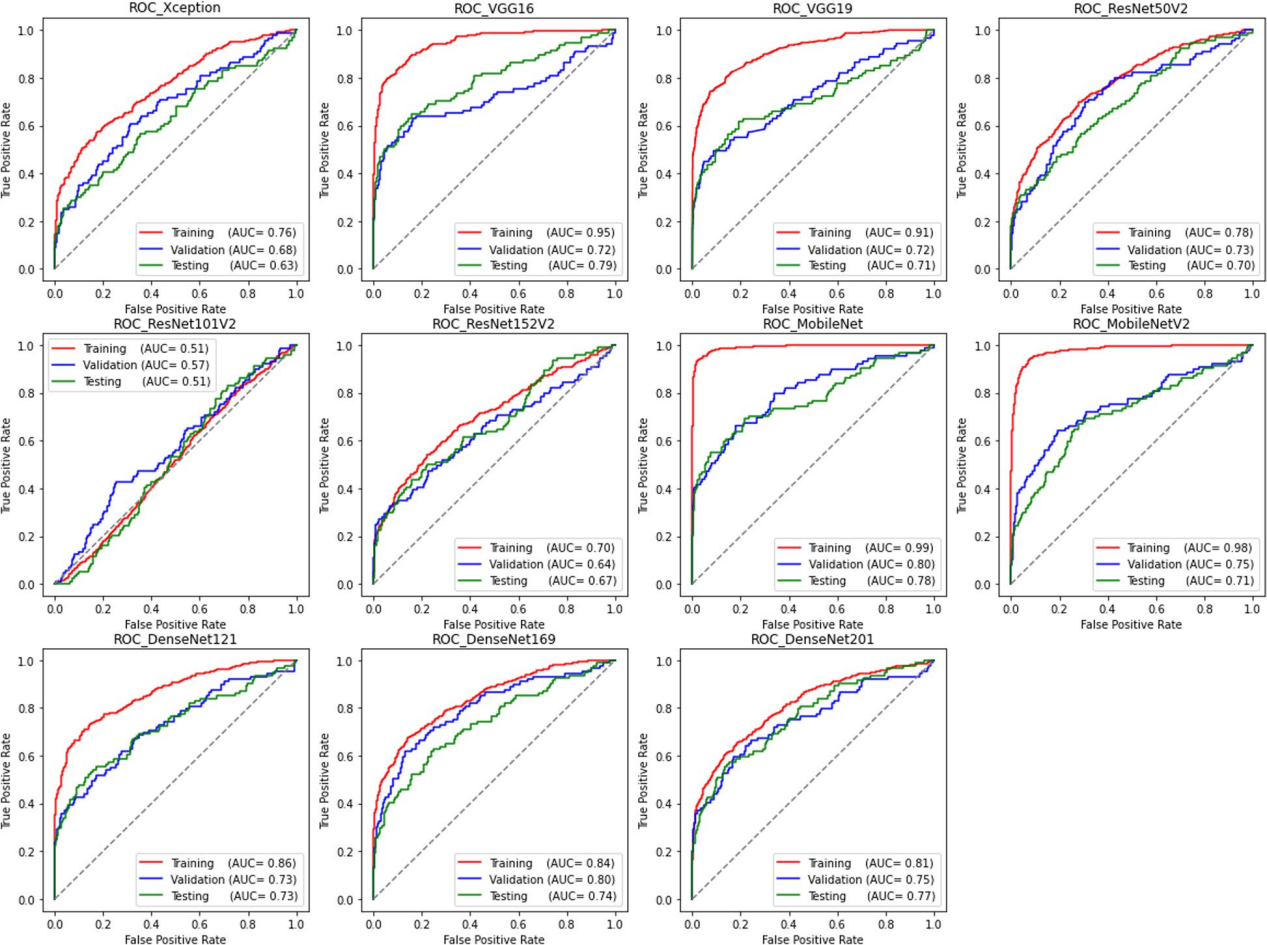
ROC curves and AUC values for each of the networks

The ROC curves and AUC values derived from fine-tuning each network on the mammographic dataset are presented in Fig. 2. The AUC values above 0.8 in the training dataset achieved by MobileNet, VGG, and DenseNet architectures indicated their good discriminatory ability between BAC and non-BAC images. The performances could be further confirmed by assessing the independent test subset, where VGG16, MobileNet, and DenseNet201 achieved the most accurate detections with AUC values of 0.79, 0.78, and 0.77, respectively. On the other hand, ResNet152V2 (0.67) and Xception (0.63) exhibited a comparatively lower performance, while ResNet101V2 demonstrated the worst result yielding an AUC of 0.51, close to a random chance classifier. Considering the convergence failure of ResNet101V2 also on the training and validation subsets, the network was eliminated from further analysis.

Table 2 reports the quantitative prediction results of the networks at their optimal operating point. Among the models tested, VGG16 (0.53), MobileNet (0.51), and VGG19 (0.46) achieved the highest *F*_1_ scores, while ResNet50V2 (0.33), Xception (0.31), and ResNet152V2 (0.29) placed at the bottom. In terms of true-positive detections, VGG16 ranked first correctly identifying 47/94 BAC images in the testing subset, higher than VGG19 and MobileNet each with 38/94 and 34/94 correct BAC detections. The architecture characteristics and the computational loads are summarized in Table 3. In general, fine-tuning each epoch of the pre-trained models on our mammographic dataset took between 241 s for lightweight MobileNet to 271 s for ResNet152V2 with the highest total number of parameters (around 59.5 million).

**Table 2:** Classification performances of the fine-tuned models

	**Training**					**Validation**					**Testing**				
	TN	TP	FN	FP	F1	TN	TP	FN	FP	F1	TN	TP	FN	FP	F1
Xception	1,192	146	252	50	0.77	772	22	67	27	0.32	767	27	67	55	0.31
VGG16	1,219	260	138	23	0.76	762	44	45	37	0.52	785	47	47	37	0.53
VGG19	1,216	237	161	26	0.51	761	40	49	38	0.48	787	38	56	35	0.46
ResNet50V2	1,209	125	273	33	0.61	784	22	67	15	0.35	801	23	71	21	0.33
ResNet152V2	1,225	83	315	17	0.33	791	22	67	8	0.37	809	18	76	13	0.29
MobileNet	1,242	247	151	0	0.62	793	36	53	6	0.55	817	34	60	5	0.51
MobileNetV2	1,232	280	118	10	0.45	778	34	55	21	0.47	781	27	67	41	0.33
DenseNet121	1,215	187	211	27	0.61	777	32	57	22	0.45	800	30	64	22	0.41
DenseNet169	1,196	199	199	46	0.49	763	37	52	36	0.46	784	34	60	38	0.41
DenseNet201	1,227	141	257	15	0.81	790	32	57	9	0.49	807	26	68	15	0.39

*TN* True negative, *TP* True positive, *FN* False negative, *FP* False positive, *F1 F*_1_ score

**Table 3:** Comparison of the deployed network characteristics

Network	Depth	Number of parameters (10^6^)		Model size (MB)	Training time (s)/epoch (s)	Testing time (ms)/image
		Total	Trainable			
Xception	36	22.04	4.34	117	251.6	39.4
VGG16	16	15.11	2.75	78.7	255.2	31.2
VGG19	19	20.42	2.75	99	262.6	38.4
ResNet50V2	50	24.74	2.23	111	242.5	28.0
ResNet152V2	152	59.51	2.23	245	271.2	61.8
MobileNet	28	3.88	1.71	28.1	241.1	15.9
MobileNetV2	53	3.04	1.20	21.2	245.9	18.9
DenseNet121	121	7.69	0.69	35.8	246.1	30.7
DenseNet169	169	13.62	1.02	61.4	249.3	39.3
DenseNet201	201	19.43	1.16	84.8	261.4	47.8

### BAC quantification

**Fig. 3:**
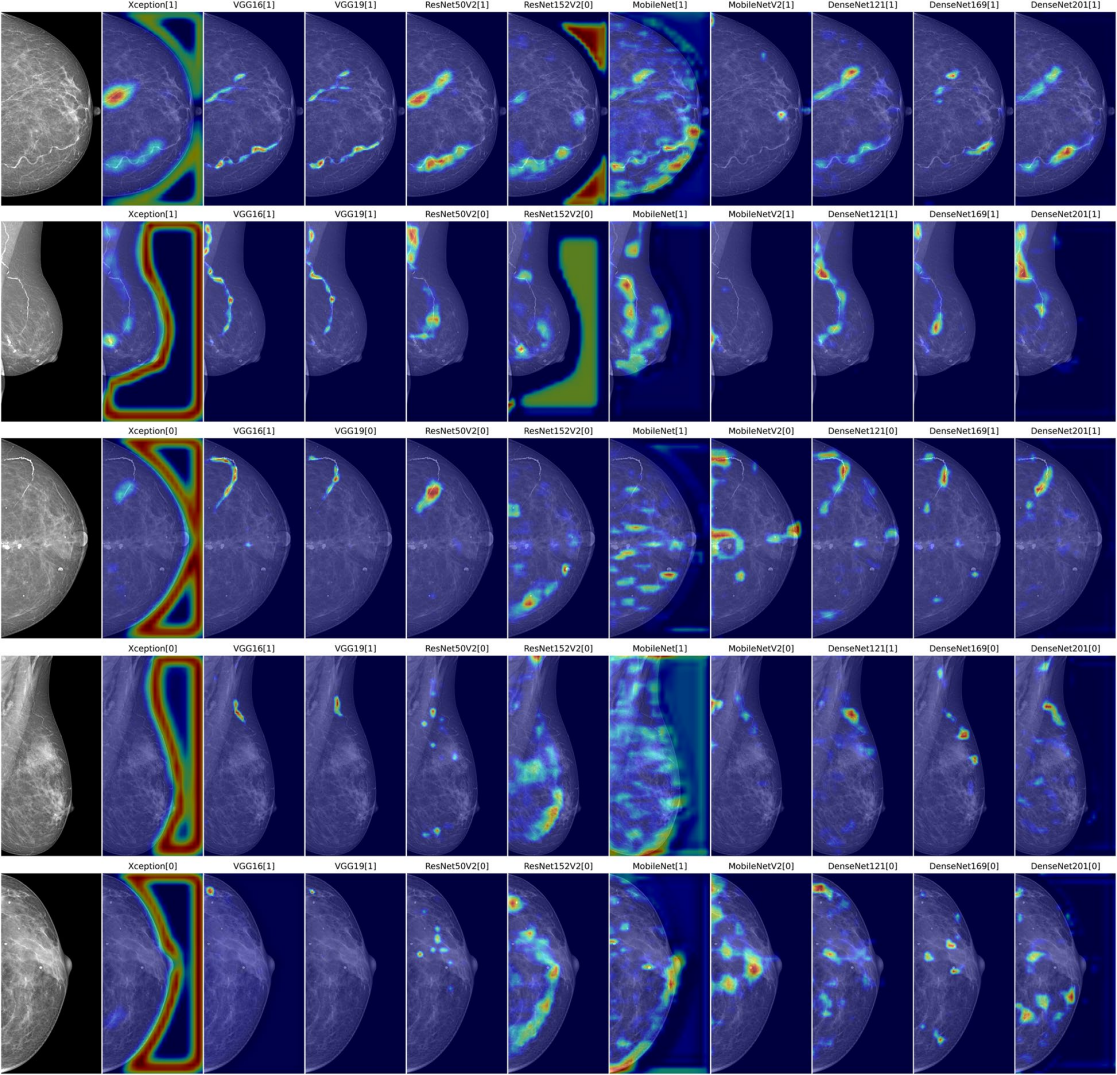
From left to right: original images (cropped to minimize the background), and examples of Grad-CAM++ heatmaps with the binary predicted labels (BAC:1 and non-BAC:0) generated from Xception, VGG16, VGG19, ResNet50V2, ResNet152V2, MobileNet, MobileNetV2, DenseNet121, DenseNet169, and DenseNet201. ResNet101V2 was excluded from the analysis due to its limited ability to effectively learn BAC features

**Fig. 4:**
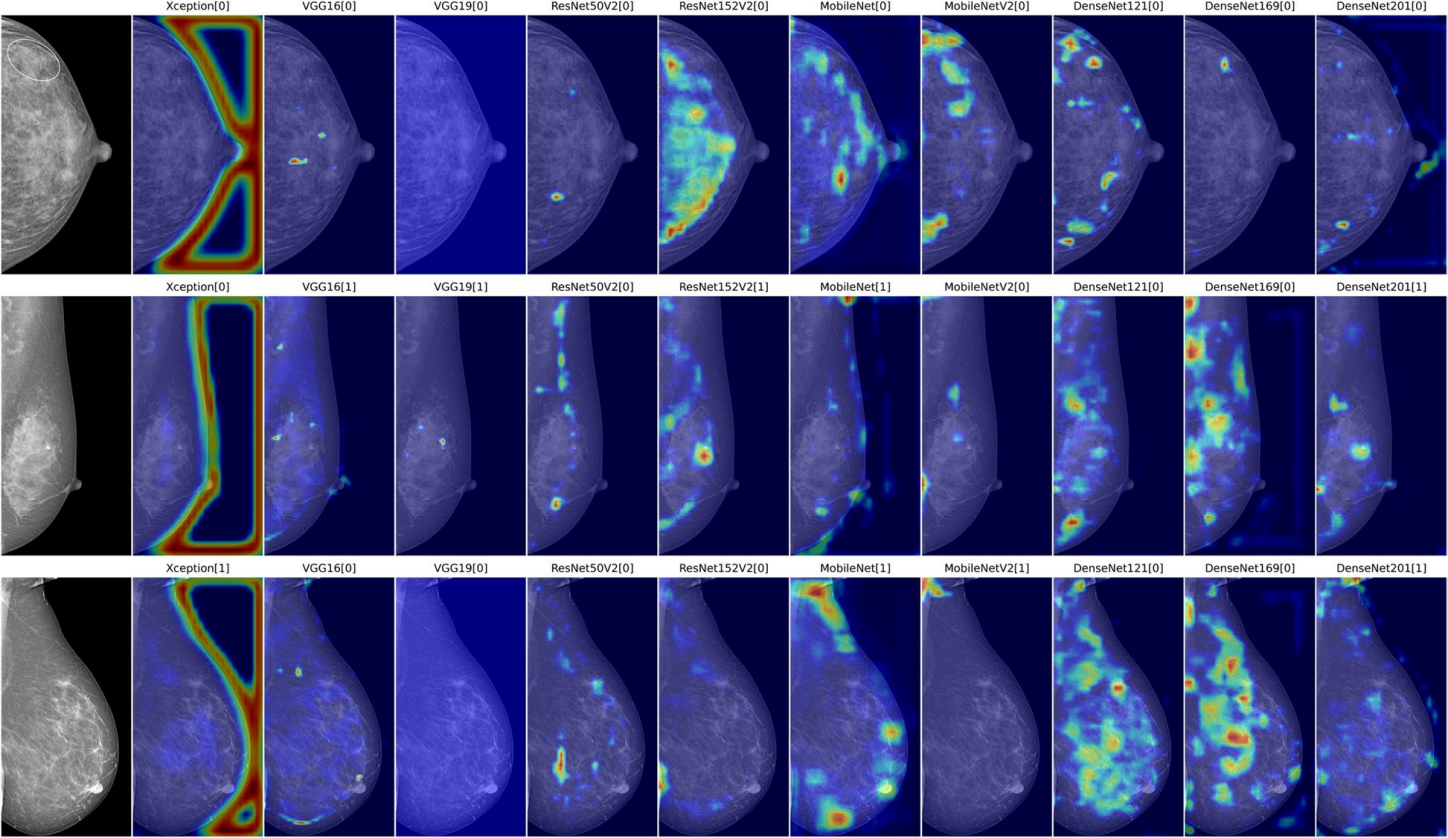
Examples of misclassifications. From top to bottom: a positive case with minor BAC concealed under the dense breast tissue (circle) misclassified as negative, and two negative cases with benign calcifications and skinfolds mistaken as BAC by some CNNs

Several examples of the Grad-CAM++ heatmaps generated from image-level ground truth are presented in Fig. 3, for an intuitive comparison of the best performances within various burdens of BAC. The localization maps mainly emphasized the regions of BAC, while de-emphasizing the overall breast with varying extent of precision. Among them, the heatmaps created by the VGG architecture explicitly outperformed those by the others in the majority of examples and provided discriminative image regions of interest that could accurately localize the area related to BAC with finer-grained details. Additional examples of wrong predictions are presented in Fig. 4. A visual assessment of the false-negative detections revealed that variables such as dense tissue or faint BAC affected the models’ accuracy in predicting the presence of BAC, but no consistent patterns were observed across different CNNs in the false positives.

**Fig. 5:**
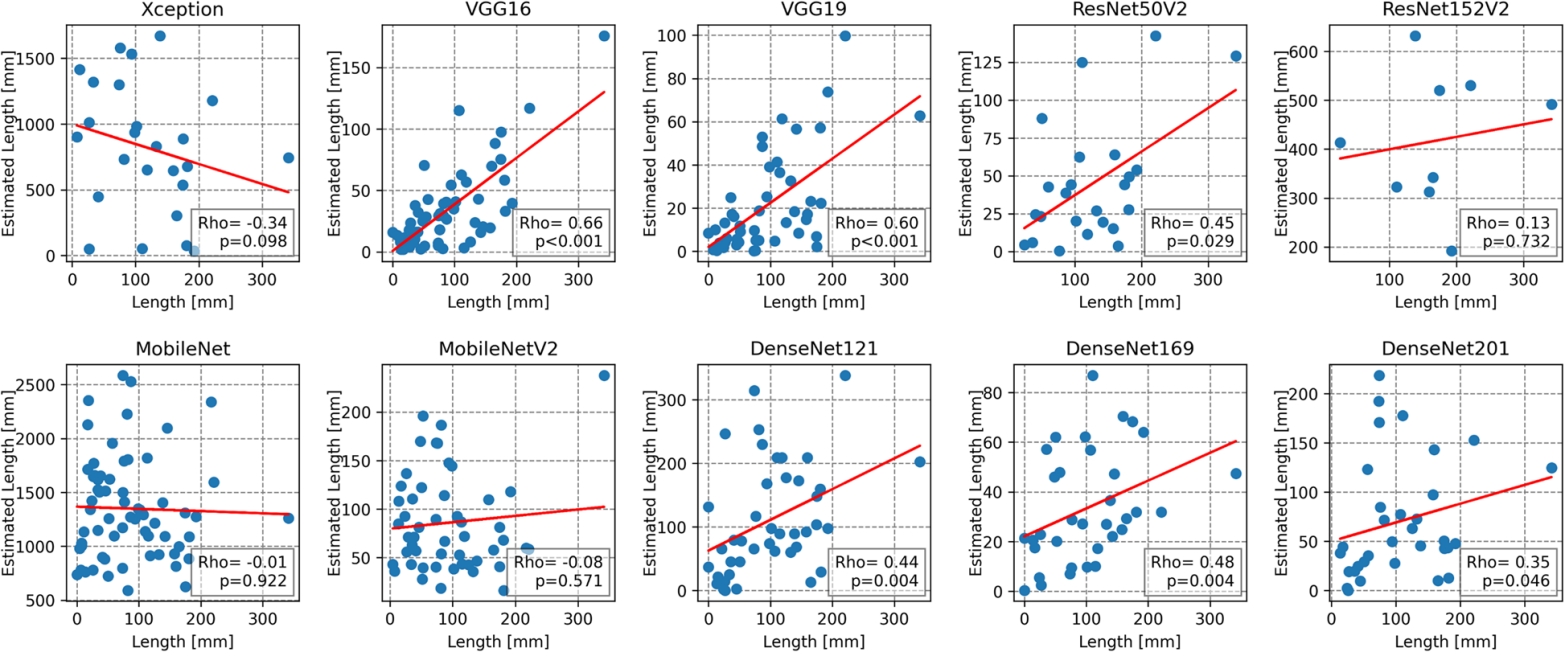
Scatterplots comparing the estimated BAC length (*y*-axis) and the manual length measurements (*x*-axis) in a subgroup of 56 women with 112 MLO views (red line, linear regression). Key statistics, including Spearman’s rank correlation coefficient (rho) and *p*-value (*p*), are provided in the lower right corner of each plot

The superiority of the VGG16 architecture in estimating the BAC region was further supported by Spearman’s rank correlation analysis (Spearman *ρ* = 0.68, *p* < 0.001), performed in a subgroup of 56 exams comprising 94 BAC out of 112 total views (Fig. 5). Meanwhile, the MobileNet ability to accurately visualize BAC areas within the images appeared inadequate and showed a poor correlation with the manually measured length, despite the good quantitative classification results.

## Discussion

In this work, we implemented different pretrained convolutional neural networks of varying depths and explored their performances for the automatic detection of BAC, a mammographic finding not related to breast cancer, which has been identified as a women-specific biomarker of cardiovascular risk. The performance ranking of the CNNs on the mammography dataset revealed that increasing depth and complexity may not necessarily improve the classification outcomes, as the best results were obtained by using relatively shallow models like VGG and MobileNet architectures in terms of higher AUC-ROC values. The highest *F*_1_ score and best visual explanation have been obtained by VGG16. When a biomarker like BAC is under consideration, these results play in favor of lightweight models being implemented quickly and efficiently even with limited hardware resources.

The use of AI networks, particularly DL-based approaches, has been explored in several studies as a solution to overcome the intrinsic limitations of manual BAC assessments [23–25]. Nonetheless, they predominantly relied on pixel-level segmentation, demanding meticulous manual annotation and often subject to observer variability. Therefore, the current study addressed the BAC classification problem based on a recently developed transfer learning-based weakly supervised framework that allows for the estimation of calcified regions using only image-level annotations, thus further reducing operator dependency and radiologists’ workload [28]. The shift toward transfer learning as a potential solution to the data scarcity problem, leveraged previously acquired knowledge of a well-established CNN network from large annotated open-source datasets and efficiently fine-tuned the relevant learned features for the specific BAC classification task at hand, rather than training from scratch [26–28].

According to our findings, VGG16, MobileNet, and DenseNet201 performed as the most promising CNNs for accurate BAC detections with superior performances among the others tested. In this setting, the depth and complexity of the neural networks do not necessarily guarantee superior performance in classifying mammography images. Both VGG16 and MobileNet are relatively shallow networks. VGG16 is characterized by a straightforward sequential architecture with small 3 × 3 convolutional filters, allowing more focused learning of relevant features, effective in various computer vision tasks [34]. MobileNet uses depth-wise separable convolutions that reduce the overall number of parameters, making it a lightweight and efficient model for mobile and embedded vision applications [36]. The other tested architectures, such as Xception and ResNetV2 [33, 35], are also recognized for their efficacy in attaining state-of-the-art results, though their performances may be influenced by the specific characteristics of the dataset and task at hand. The superiority of smaller networks to their deeper counterparts, when it comes to medical dataset often with a limited number of samples, has also been observed in some other studies exploring DL techniques for a wide variety of diagnostic medical imaging applications such as chest x-ray classification or breast cancer diagnosis [48, 49].

The qualitative assessment of performances through generalized Grad-CAM complemented the quantitative analysis based on the AUC-ROC and *F*_1_ score metrics. Notably, the inherent simplicity and uniformity of the VGG16 architecture facilitated a more precise representation of the distinctive patterns associated with BAC on mammograms. These heatmaps hold potential for application in weakly supervised segmentation, as we previously elaborated in [28], wherein BAC localization is achieved by a CNN trained only on image-level labels, without requiring pixel-by-pixel ground truth annotations. Consequently, an estimation of the BAC burden, as a by-product of the automatic detection framework, could be obtained by using simple thresholding and segmenting out the most intense pixels of the Grad-CAM++ heatmaps which encapsulated calcified areas of the original image. Furthermore, this visual approach introduces the prospect of integrating human expertise into the decision-making loop, as clinicians could contribute their insights to further refine the segmentation or improve the CNN model based on the visual cues provided by the heatmaps.

The comparability of our method and the other cited research may be limited as detailed BAC segmentations were mostly used to evaluate the outcomes [23–25]. The original study that proposed the novel weakly supervised BAC detection framework, achieved a promising performance by fine-tuning VGG16 with an AUC-ROC of 0.94 in the testing subset and a strong correlation with manual BAC measurements (Spearman *ρ* = 0.88, *p* < 0.001) [28], surpassing all models in our analysis. Indeed, in the current experiment, a uniform transfer learning strategy followed by a harmonized hyperparameter set was adopted across all networks, which were probably not selected as precisely as in [28], since our priority was comparing architectures rather than optimizing each model. Furthermore, all models were evaluated on an independent testing subset reflecting real-world BAC prevalence of around 12%, as in the original research [28]. This realistic imbalanced subset ensures the CNNs’ stability and robustness for future studies with BAC as the minority class, in contrast to the previous research that predominantly included BAC exams, risking model overfitting.

The present study has some limitations. First, the dataset included in this retrospective analysis was obtained from a single imaging center using two mammographic devices by the same manufacturer, which may introduce potential biases and constrain the generalizability of the findings. Second, while using a uniform training strategy across all neural network architectures enabled a fair comparison, it may limit the full potential of each model. Further research is warranted to explore customized configurations tailored to the unique characteristics of each architecture to exploit their maximum capabilities and optimize their performances. Third, the chosen metrics for performance evaluation provide robust insights, yet the clinical relevance of these metrics to real-world patient outcomes remains an area for future investigation. Lastly, we did not compare the diagnostic performances of the different models to that of a radiologist; however, this kind of evaluation was beyond the aims of the current work and will be addressed in future research.

In conclusion, this study demonstrated the efficacy of employing deep transfer learning-based approaches for BAC on mammograms, where networks such as VGG16 and MobileNet outperformed their deeper more complex counterparts. The competitive performance and notable computational efficiency of simpler networks highlighted the viability of adopting such models in clinical settings with substantial savings in both time and resources. Our extensive experiment and evaluations, both quantitative and qualitative, could provide valuable insights for researchers in selecting exemplary network architectures for automatic BAC detection and developing efficient tools for early CVD risk stratification in asymptomatic women. Further research is required to address the limitations and validate the models using a larger diverse study population, ultimately paving the way for integrating the models into clinical practices without any time loss for radiologists and fostering awareness of women’s cardiovascular health in the context of widespread mammographic screening programs. Conversely, the use of mammographic images for cardiovascular risk stratification could be an added new motivation for participation in screening mammography programs, thus reinforcing its value also for secondary prevention of breast cancer in the female population [8]. As the field continues to evolve, a balance between diagnostic accuracy, computational efficiency, and real-world applicability will be crucial.

## Data Availability

The full database is published in the Zenodo repository for data sharing [https://zenodo.org/records/11571849]. Dataset used in a previously published work: Mobini, N., Codari, M., Riva, F. et al. Detection and quantification of breast arterial calcifications on mammograms: a deep learning approach. Eur Radiol 33, 6746–6755 (2023). 10.1007/s00330-023-09668-z.

## Abbreviations

AI: Artificial intelligence
AUC-ROC: Area under the receiver operating characteristic curve
BAC: Breast arterial calcifications
CNN: Convolutional neural network
CVD: Cardiovascular diseases
DenseNet: Dense convolutional network
DL: Deep learning
FN: False negative
FP: False positive
Grad-CAM++: Generalized gradient-weighted class activation mapping
IQR: Interquartile range
MLO: Medio-lateral oblique
ResNet: Residual network
TN: True negative
TP: True positive
VGG: Visual Geometry Group
Xception: Extreme Inception
